# Calpain-mediated cleavage of DARPP-32 in Alzheimer’s disease

**DOI:** 10.1111/acel.12374

**Published:** 2015-07-14

**Authors:** Kwangmin Cho, Mi-Hyang Cho, Jung-Han Seo, Jongjin Peak, Kyoung-Hye Kong, Seung-Yong Yoon, Dong-Hou Kim

**Affiliations:** 1 Alzheimer’s Disease Experts Lab (ADEL), Asan Medical Center, University of Ulsan College of MedicineSeoul, Korea; 2 Department of Brain Science, University of Ulsan College of MedicineSeoul, Korea; 3 Bio-Medical Institute of Technology (BMIT), University of Ulsan College of MedicineSeoul, Korea; 4 Cell Dysfunction Research Center (CDRC), University of Ulsan College of MedicineSeoul, Korea

**Keywords:** Alzheimer’s disease, calpain, cAMP-response element-binding protein, DARPP-32, okadaic acid, protein kinase A

## Abstract

Toxicity induced by aberrant protein aggregates in Alzheimer’s disease (AD) causes synaptic disconnection and concomitant progressive neurodegeneration that eventually impair cognitive function. cAMP-response element-binding protein (CREB) is a transcription factor involved in the molecular switch that converts short-term to long-term memory. Although disturbances in CREB function have been suggested to cause memory deficits in both AD and AD animal models, the mechanism of CREB dysfunction is still unclear. Here, we show that the dopamine- and cAMP-regulated phosphoprotein 32 kDa (DARPP-32), a key inhibitor of protein phosphate-1 (PP-1) that regulates CREB phosphorylation, is cleaved by activated calpain in both AD brains and neuronal cells treated with amyloid-β or okadaic acid, a protein phosphatase-2A inhibitor that induces tau hyperphosphorylation and neuronal death. We found that DARPP-32 is mainly cleaved at Thr^153^ by calpain and that this cleavage of DARPP-32 reduces CREB phosphorylation via loss of its inhibitory function on PP1. Our results suggest a novel mechanism of DARPP-32–CREB signalling dysregulation in AD.

## Introduction

Alzheimer’s disease (AD), a very common neurodegenerative disease, is characterized by progressive impairment of cognitive function and memory formation. Pathological signalling in AD is largely mediated by two major characteristic components, neurofibrillary tangles and senile plaques (Gomez-Isla *et al*., 1997). Extracellular plaques are primarily composed of amyloid-β (Aβ) peptides, which are derived from amyloid precursor protein via proteolytic processing. Neurofibrillary tangles are formed by intraneuronal accumulation of paired helical filaments composed of abnormally hyperphosphorylated tau protein (Grundke-Iqbal *et al*., 1986).

cAMP-response element-binding protein (CREB), a ubiquitous transcription factor, is a key molecule for learning and memory and a core component of the molecular switch that converts short-term to long-term memory (Barco *et al*., 2003). Various stimuli, such as NMDA receptor activation and increased Ca^2+^ internalization, induce CREB phosphorylation, which results in the activation of the expression of many genes required for synaptic plasticity and memory formation. CREB activation is regulated by phosphorylation/dephosphorylation at serine 133, which is mediated by various kinases and phosphatases. The major kinases mediating CREB phosphorylation are mitogen-activated protein kinase (MAPK), Ca^2+^/calmodulin-dependent protein kinase II/IV (CaMKII/IV) and cAMP-dependent protein kinase A (PKA). Protein phosphatase-1 (PP-1) mainly inactivates CREB by mediating its dephosphorylation. A disturbance in CREB function has been suggested to cause memory loss in AD brains and AD animal models (Yamamoto-Sasaki *et al*., 1999; Gong *et al*., 2004; Puzzo *et al*., 2005). Aβ is associated with reduced p-CREB levels caused by the inhibition of PKA activity (Vitolo *et al*., 2002). Aβ also reduces CREB phosphorylation by decreasing NMDA receptor levels in primary neurons (Ma *et al*., 2007). In the signalling cascade downstream of PKA to CREB activation, dopamine- and cAMP-regulated phosphoprotein 32 kDa [DARPP-32, also called PP1 regulatory subunit 1B (PPP1R1B)] is one of the key molecules regulating the activation state of the PKA–CREB pathway (Svenningsson *et al*., 2004). When the cAMP level increases, PKA is activated and phosphorylates DARPP-32 at Thr34, which inhibits PP1 and increases CREB phosphorylation (Hemmings *et al*., 1990). Although it is important as a key molecule in the CREB activation pathway, DARPP-32 has not been investigated in AD brains or other AD models.

In this study, we investigated the association between DARPP-32 and CREB malfunction in AD pathology and found cleavage of DARPP-32 in AD brain tissue and neuronal cells treated with Aβ or okadaic acid (OA), a protein phosphatase-2A (PP2A) inhibitor that induces tau hyperphosphorylation and neuronal death *in vitro* (Yoon *et al*., 2012). Interestingly, calpain inhibitors reversed DARPP-32 cleavage in OA-treated primary neurons and recombinant DARPP-32 protein was mainly cleaved by calpain *in vitro*. The results suggest that calpain-induced DARPP-32 cleavage and PP1 activation may contribute to the impairment of the PKA–CREB pathway in AD pathogenesis.

## Results

### Different sizes of DARPP-32 proteins in AD brains

**Fig 1:**
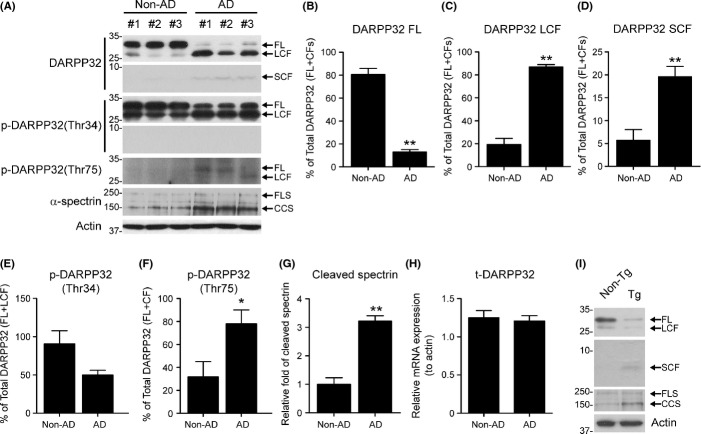
Decreased level of DARPP-32 in Alzheimer’s disease (AD) brain. (A) The expression of DARPP-32 in AD and non-AD brains was analysed by Western blotting with antibodies to DARPP-32, phosphorylated DARPP-32 (pThr34, pThr75) and α-spectrin. FL, full-length DARPP-32; LCF, long cleavage fragment of DARPP-32; SCF, short cleavage fragment of DARPP-32; FLS, full-length α-spectrin; CCS, calpain-cleaved α-spectrin. (B–G) Representative graphs showing quantification of the levels of FL DARPP-32 (B), DARPP-32 fragments (C, D), phosphorylated DARPP-32 (E, F) and calpain-cleaved α-spectrin (G) shown in (A). The expression levels of all proteins were normalized with respect to that of total DARPP-32 (B–F). Data are presented as the mean ± SEM (*P < 0.05, **P < 0.001). (H) mRNA expression of t-DARPP-32 in AD brains (*n* = 8) and age- and sex-matched control brains (*n* = 7) (_)Table1) analysed by qPCR. The mRNA level of t-DARPP-32 was normalized by comparison with actin. (I) Western blot of DARPP-32 in APP/PS1 mice (Tg, 12 months of age) and control mice (non-Tg, 15 months of age).

To investigate whether DARPP-32 is involved in the pathogenesis of AD, we compared the levels of DARPP-32 protein between control and AD patients. Interestingly, the total level of DARPP-32 protein was lower in AD brains (by ∼20%) than in control groups (Fig.1A,B). We also found increases in two smaller (∼28 and ∼4 kDa) alternative forms of DARPP-32 in AD brains (Fig.1A,C–D). These alternative DARPP-32 proteins were also found in APP/PS1 mouse brain (Fig.1I). An alternative spliced form of DARPP-32, t-DARPP-32, has previously been reported (El-Rifai *et al*., 2002). Because the alternative larger DARPP-32 form (∼28 kDa) was similar in size to previously identified t-DARPP-32, we examined the phosphorylation status at Thr34 in this alternative form to compare it with that of t-DARPP-32, which lacks the Thr34 phosphorylation site (El-Rifai *et al*., 2002). The larger DARPP-32 form (∼28 kDa) was phosphorylated at Thr34, and this phosphorylation was unchanged in AD patients (Fig.1A,E). In accordance with this result, the mRNA level of t-DARPP-32 in AD brains was comparable with that of the control group (Fig.1H). Interestingly, phosphorylation at Thr75 in DARPP-32 WT and its fragments, which results in DARPP-32 inactivation (Bibb *et al*., 1999), was increased in AD brain (Fig.1F). These results showed that different cleavage products of DARPP-32 exist in human AD brains.

### Lower DARPP-32 level in OA- and Aβ-treated neurons

**Fig 2:**
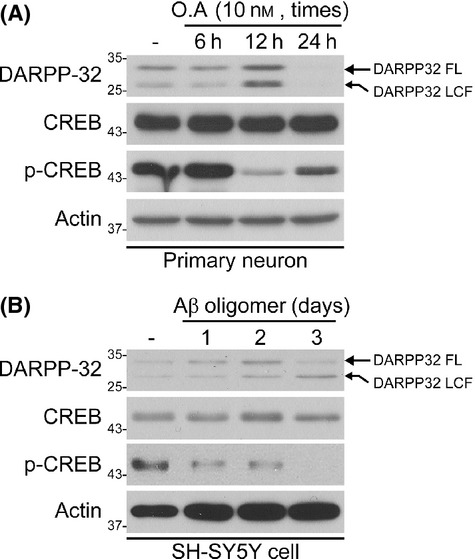
Decreased DARPP-32 and cAMP-response element-binding protein phosphorylation in okadaic acid (OA)-treated primary neurons or Aβ-treated SH-SY5Y cells. (A, B) Representative Western blots of DARPP-32 in OA-treated primary neurons and Aβ-treated SH-SY5Y cells. Primary neurons and SH-SY5Y cells were treated with OA (A) or Aβ (B) for the indicated times. The expression level of DARPP-32 was determined by Western blot analysis.

To understand the mechanism of the decrease in the DARPP-32 level in AD, we examined the DARPP-32 protein level in primary neurons and SH-SY5Y cells treated with OA or Aβ peptides, which have previously been used to mimic AD pathology in cell culture models. Consistent with our earlier results (Fig.1), DARPP-32 protein was cleaved to a 28-kDa fragment that was smaller and less abundant in both AD cell models (Fig.2). We also found a decrease in CREB phosphorylation under the same conditions (Fig.2). These results suggest that a lower DARPP-32 level may contribute to the impairment of PKA–CREB signalling in AD.

### Blockage of the decrease in DARPP-32 by calpain inhibitors in OA-treated neurons

**Fig 3:**
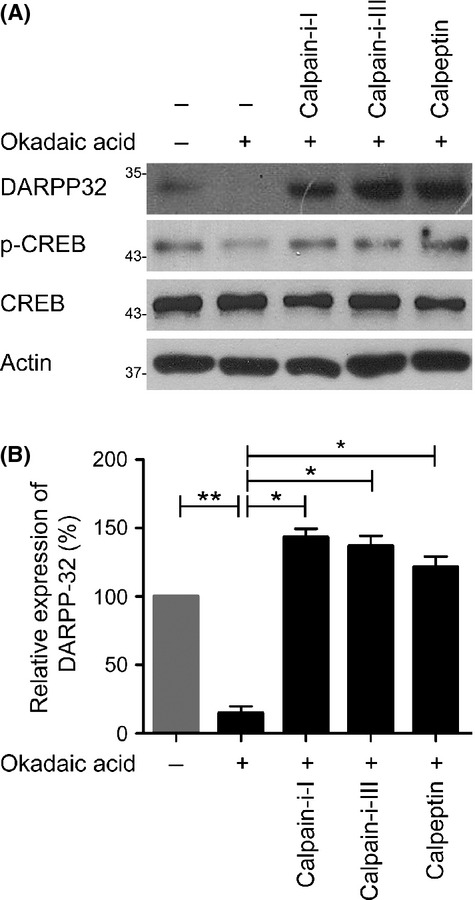
Calpain inhibition blocks the decrease in the DARPP-32 level in okadaic acid (OA)-treated primary neurons. (A) Calpain inhibitors block the OA-induced decrease in DARPP-32 in primary neurons. Primary neurons were treated with OA in the presence or absence of calpain inhibitors (calpain-i-I, calpain-i-III or calpeptin) as indicated. Lysates were analysed by Western blot. (B) Quantification of the DARPP-32 expression shown in (A). Data are presented as the mean ± SEM (*P < 0.05, **P < 0.001).

Because the aberrant DARPP-32 protein expression was not due to alternative splicing (Fig.1G), we speculated that proteolytic cleavage activity might be involved in the production of DARPP-32 protein fragments. We previously reported that some key molecules were proteolytically cleaved by activated calpain in OA-treated neurons (Yoon *et al*., 2006, 2007, 2008) and that enhanced calpain activity induced by an increase in the cytosolic calcium concentration mediated by Aβ led to cleavage of key proteins in AD. We also found that the calpain-induced α-spectrin cleavage product was present at a higher level in AD brain tissues (Fig.1A,D) and APP/PS1 Tg mouse brain (Fig.1I). To determine whether calpain is linked to DARPP-32 degradation, we examined the DARPP-32 protein level in primary neurons treated with OA, with or without various calpain inhibitors. As expected, the decrease in the DARPP-32 level caused by OA was blocked by co-incubation with calpain inhibitors (Fig.3). We also found that the decrease in CREB phosphorylation recovered under the same condition. Therefore, these results suggest that the decrease in DARPP-32 in AD is due to proteolytic cleavage by activated calpain and that calpain activity is linked to CREB signalling via DARPP-32 cleavage.

### Cleavage of recombinant DARPP-32 by calpain

**Fig 4:**
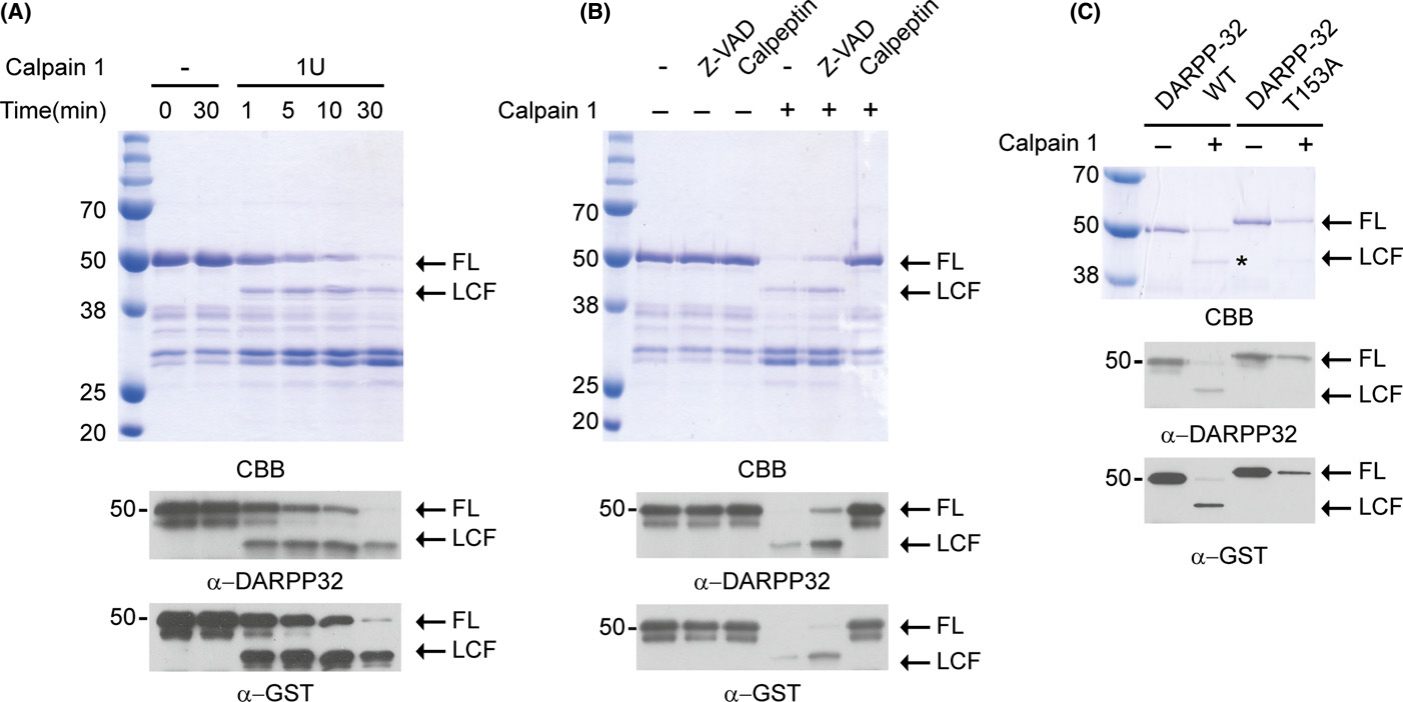
Recombinant DARPP-32 is cleaved by calpain but mutation of Thr153 blocks generation of the DARPP-32 fragment. (A) Cleavage of recombinant GST–DARPP-32 by calpain-1. Calpain-1 was incubated with recombinant GST–DARPP-32 at 30 °C for the indicated times. The cleaved DARPP-32 fragment [long cleavage fragment (LCF)] was visualized by Coomassie blue staining and Western blotting with antibodies to DARPP-32 and GST. (B) Calpain inhibitor blocks DARPP-32 cleavage by calpain. Recombinant DARPP-32 was incubated with calpain-1 in the presence of calpeptin or zVAD, a caspase inhibitor. The assay was performed as described in (A). (C) Thr153 of DARPP-32 is a major cleavage site. Recombinant DARPP-32 or DARPP-32 T153A mutant proteins were incubated with calpain-1. The assay was performed as described in (A).

To determine whether DARPP-32 is directly cleaved by calpain, we digested recombinant DARPP-32 with calpain-1 for various times and examined the DARPP-32 fragments by Western blot analysis and Coomassie blue staining. We detected a major cleavage product band (∼40 kDa) that showed a size that was similar to the difference in size between the larger variant and the full-length form of DARPP-32 in AD brain and AD cell models (Figs1 and 4A). Full-length GST–DARPP-32 rapidly disappeared after 30 min, but the cleavage product (GST–DARPP-32 CF) appeared at an early time (1 min) and was resistant to further calpain cleavage (Fig.4A). We confirmed that this cleavage product was processed from GST–DARPP-32 by Western blotting with both DARPP-32 and GST antibodies (Fig.4A). When the cleavage reaction mixture was incubated with calpeptin, a calpain inhibitor, the cleavage product was not generated (Fig.4B).

To identify the calpain cleavage site of DARPP-32, we used a computational program (gps-ccd) (Liu *et al*., 2011) that predicts potential cleavage sites. Our earlier data showed that the larger endogenous DARPP-32 cleavage product contained the Thr34 residue (Fig.1A) and that the N-terminal GST tag of the recombinant DARPP-32 cleavage product remained after calpain cleavage (Fig.4A,B). Because these results suggested that the DARPP-32 cleavage site is likely to be near the C-terminal region, we selected Thr153 as a potential cleavage site and mutated it to alanine (DARPP-32 T153A). As shown in Fig.4C, DARPP-32 T153A was resistant to calpain cleavage, suggesting that this site may be the primary calpain cleavage site in DARPP-32.

### Expression of DARPP-32 or DARPP-32 T153A protects primary neurons and SH-SY5Y cells from Aβ toxicity

**Fig 5:**
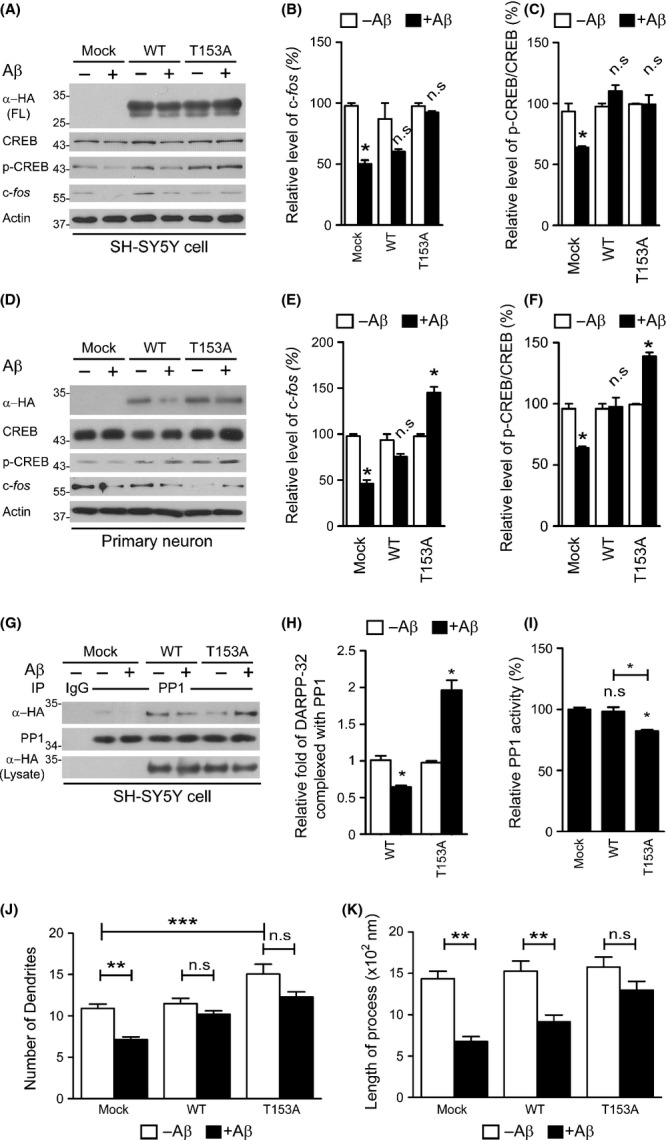
Expression of DARPP-32 T153A increases neuronal survival and cAMP-response element-binding protein (CREB) phosphorylation in Aβ-treated primary neurons and SH-SY5Y cells. (A–F) SH-SY5Y cells (A–C) or primary neuron (D–F) expressing DARPP-32 WT or the T153A mutant was treated with Aβ oligomer (1 μm) for 24 h. Cell lysates were analysed by Western blot to determine the levels of p-CREB and c-*fos* expression. (B, C, E, F) Quantification of p-CREB and c-*fos* expression shown in (A, D). Data are presented as the mean ± SEM (*P < 0.05). (G) Cell lysates were prepared from SH-SY5Y cells expressing DARPP-32 WT or the T153A mutant under the same conditions as in (A) and were subjected to immunoprecipitation with anti-PP1 antibody followed by Western blot. (H) Quantification of DARPP-32 WT and its T153 mutant complexed with PP1 shown in (G). Data are presented as the mean ± SEM (*P < 0.05). (I) After isolation of PP1 under the same condition mentioned above (G), PP1 was incubated with DiFMUP, a fluorogenic PP1-specific substrate, for 30 min at RT. After incubation, fluorescence from the reaction mixture was measured. Data are presented as the mean ± SEM (**P < 0.001, *P < 0.05). (J, K) Primary mouse neurons transfected with cDNA for DARPP-32 WT or the T153A mutant (these constructs also separately express GFP) were incubated with the Aβ oligomer (1 μm) for 24 h. The dendrite number and length of GFP-positive neurons (*n* = 100) were measured and are presented as bar graphs using data from three independent experiments. Data are presented as the mean ± SEM (**P < 0.001, ***P < 0.005).

Because DARPP-32 regulates phosphorylation of CREB through PP-1 inhibition, we speculated that DARPP-32 cleavage would result in dysregulated CREB signalling and weaken the protective effect of CREB in neurons against toxicity. To verify this hypothesis, we first investigated the change in CREB signalling in Aβ-treated SH-SY5Y cells expressing DARPP-32 WT or the DARPP-32 T153A mutant. Aβ treatment reduced CREB phosphorylation and the expression of *c-fos*, its downstream target gene (Fig.5A–C). Interestingly, DARPP-32 WT or the T153A mutant rescued CREB phosphorylation and c-*fos* expression (Fig.5A–C). In the same experiment with primary neurons, we found that Aβ treatment induced a decrease in the full-length DARPP-32 WT expression level, whereas no such change was detectable for DARPP-32 T153A, confirming that the T153A mutation prevents the cleavage of DARPP-32 in primary neurons. Dysregulation of CREB signalling by DARPP-32 cleavage was confirmed in primary neurons under the same condition (Fig.5D–F), suggesting that loss of DARPP-32 leads to dysregulation of CREB signalling. To investigate the detailed mechanism, we first examined the interaction between PP1 and DARPP-32 WT or the T153A mutant. It is already known that DARPP-32 inhibits PP1 activity by directly interacting with PP1 (Huang *et al*., 1999). Interestingly, Aβ treatment inhibited the interaction between PP1 and DARPP-32 WT, while the DARPP-32 T153A mutant interacted more strongly with PP1 (Fig.5G,H). Because this interaction is critical for the inhibitory function of DARPP-32 on PP1, it is expected that differences in the strength of the interaction between PP1 and DARPP-32 WT or the DARPP-32 T153A mutant will lead to differences in PP1 activity. Consistent with this expectation, DARPP-32 T153A mutant expression resulted in strong PP1 inhibition (Fig.5I). Finally, we examined for recovery of CREB signalling after the expression of DARPP-32 WT or the T153A mutant in Aβ-treated primary neurons. Consistent with our previous data (Fig.5A–I), Aβ treatment inhibited dendrite growth of mock-transfected primary neurons (Fig.5J,K). This inhibition was blocked by the expression of DARPP-32 WT or the T153A mutant (Fig.5J,K). DARPP-32 T153A mutant expression also increased dendrite growth in the absence of Aβ (Fig.5J), suggesting that the blockage of DARPP-32 cleavage protects neurons from Aβ toxicity by maintaining CREB signalling for neuronal growth.

## Discussion

**Fig 6:**
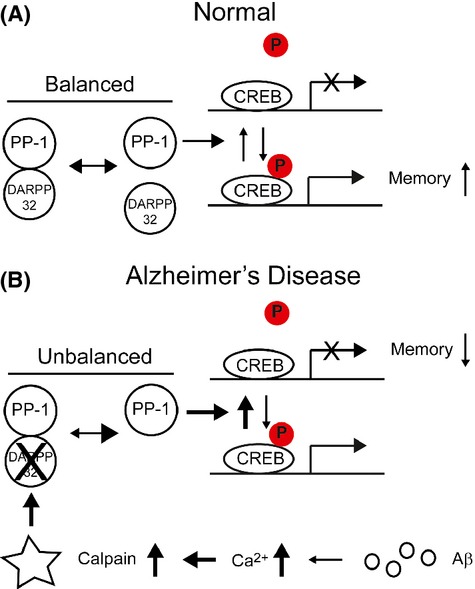
Hypothetical model for downregulation of DARPP-32–cAMP-response element-binding protein (CREB) signalling in Alzheimer’s disease (AD). (A) In normal conditions, CREB dephosphorylation is regulated by a balance between free PP1 and PP1 complexed with DARPP-32. (B) In AD, Aβ increases the cytosolic calcium concentration, leading to the activation of calpain and cleavage of DARPP-32. An imbalance between the level of PP1 bound to DARPP-32 and that of free PP1 increases CREB dephosphorylation, which in turn results in a subsequent decrease in the transcription of genes involved in memory.

In the present study, we observed a lower level of full-length DARPP-32 and two new DARPP-32 fragments (∼28 and ∼4 kDa) in AD patients and APP/PS1-Tg mouse brain samples. DARPP-32 was cleaved by calpain and the cleaved products lost their ability to regulate CREB phosphorylation. The expression of DARPP-32 T153A, a noncleavable mutant, increased CREB activity and protected neurons from Aβ toxicity. On the basis of our findings, we propose a novel pathological mechanism for AD involving disturbed CREB signalling caused by proteolytic cleavage of DARPP-32 (Fig.6). Under normal conditions, dephosphorylation of CREB is regulated by the balance between free PP1 and PP1 complexed with DARPP-32. However, an increase in Aβ initiates a series of reactions that lead to a higher intracellular calcium level, resulting in calpain activation and DARPP-32 cleavage. Degradation of DARPP-32 induces an increase in the level of free PP1 by destabilizing the PP1–DARPP-32 complex and dysregulation of CREB signalling, resulting in synaptic dysfunction and cognitive impairment.

A major finding of the current study is that calpain cleaves DARPP-32 and induces CREB dephosphorylation, which ultimately result in synaptic abnormalities. Calpain is an abundant cytoplasmic cysteine protease that can cleave many intracellular signalling and structural proteins. Pathological overactivation of calpain mediates abnormal degradation of many proteins and can lead to cell death. Calpain overactivation has also been reported in AD and has been proposed to play an important role in both cytoskeletal pathogenesis and neurodegeneration (Saito *et al*., 1993; Adamec *et al*., 2002; Higuchi *et al*., 2012). Excessive calpain activation is also responsible for the truncation of CREB, PP2B and PKA-R, which modulate CREB signalling (Liu *et al*., 2005; Liang *et al*., 2007; Jin *et al*., 2013). Recently, calpain inhibition has been reported to improve memory and synaptic transmission in an AD mouse model by restoring CREB phosphorylation (Trinchese *et al*., 2008). This report suggested that decreased CREB phosphorylation may be due to calpain-induced degradation of kinases phosphorylating CREB, such as PKA and CaMKII. In addition, the results of the current study show that CREB dephosphorylation maintained by DARPP-32 is also downregulated by calpain in AD cell models (Figs5).

cAMP-response element-binding protein is a central regulator of long-lasting synaptic plasticity and is involved in plasticity and learning in many contexts (Barco *et al*., 2003). Accordingly, dysregulation of CREB activity has been implicated in various CNS disorders, including AD, Huntington’s disease, Parkinson’s disease, ischaemia and addiction (Walton & Dragunow, 2000; Nucifora *et al*., 2001; Ma *et al*., 2007; Sawamura *et al*., 2008). Aβ-mediated CREB dysfunction leads to reductions in the levels of synaptic plasticity-related genes, such as *Bdnf*, *Nr4a2* and *c-fos* (Espana *et al*., 2010). Notably, the expression of the DARPP-32 T153A mutant blocked the decrease in *c-fos* expression by restoring CREB phosphorylation (Fig.5A,B,D,E). Therefore, the results of the current study open the prospect of using dysregulated CREB phosphorylation as a target for the treatment of memory disorders in AD patients.

Previous therapeutic trials have aimed to increase the phosphorylation and kinase activity of CREB. Some natural products, including catechins (from green tea), blueberry extract and ginsenoside (from ginseng), increased CREB phosphorylation by increasing protein kinase activity (PKA, ERK1/2, RSK2, CaMKII) (Williams *et al*., 2008; Li *et al*., 2009; Zhao *et al*., 2009). In accordance with the results of the present study, developing drugs that increase DARPP-32 activity could also be a good therapeutic option for the treatment of AD. DARPP-32 has been suggested to be involved in CNS disorders, including schizophrenia, depression and Parkinson’s disease (Cash *et al*., 1987; Guitart & Nestler, 1992; Albert *et al*., 2002). We have shown here that the total level of DARPP-32 is decreased in both AD brains and OA-treated neurons (Figs1 and 2). DARPP-32 is a key molecule in the cAMP–PKA–CREB pathway, where it acts as an on–off switch depending on its phosphorylation state (Svenningsson *et al*., 2004). Thus, developing ways to increase the total level of DARPP-32 and phosphorylation at Thr34 may be an effective approach for the treatment of AD and other CNS disorders. Several molecules, including adrenergic agonists, dopaminergic agonists and antidepressants, increase DARPP-32 phosphorylation at Thr34 (Svenningsson *et al*., 2004). Here, we suggest that calpain inhibitors could be used as DARPP-32 modulators.

DARPP-32 was cleaved at Thr153 by calpain (Fig.4), and expression of the noncleavable mutant T153A inhibited the neurodegeneration induced by Aβ by restoring the CREB signalling pathway (Fig.5). In particular, Aβ negatively regulated the PP1–DARPP-32 interaction, resulting in the release of PP1 from DARPP-32 inhibition. Indeed, DARPP-32 T153A interacted more strongly with PP1 than with DARPP-32 and inhibited PP1 activity (Fig.5G–I). These findings suggest that the negative regulation of CREB signalling by Aβ occurs as a result of the liberation PP1 from the PP1–DARPP-32 complex by calpain degradation of DARPP-32. Contrary to the fast degradation of DARPP-32 fragments in primary neurons or SH-SY5Y cells (Figs2 and 5), DARPP-32 fragments (both short and long cleavage fragments) accumulated in AD human brains and APP/PS Tg mouse brain (Fig.1). Moreover, these fragments exhibited increased phosphorylation at Thr75, which causes inactivation of DARPP-32 (Fig.1A,F), indicating that these fragments may be harmful to neurons and that further studies are needed to identify their role(s) in AD pathology.

In conclusion, the present study is the first to show that DARPP-32 is a substrate for calpain cleavage in AD and that its cleavage results in impaired CREB signalling. This finding further supports the need to develop calpain inhibitors as AD therapeutics.

## Materials and methods

### Human brain tissues and APP/PS1-Tg mouse brain tissue

Medial temporal gyri from eight AD patients and seven age- and sex-matched controls were provided by the Netherlands Brain Bank (Table1). Pathological staging of AD was based on the Braak staging system (Braak & Braak, 1991). Whole brain tissue from same aged (12 month) control and APP/PS1 mice was analysed by Western blot.

**Table 1:** Human medial temporal gyrus samples used in this study

Diagnosis	Sex	Age	Braak	Amyloid	PMD	pH	Weight
Alzheimer’s disease	M	85	5	C	07:10	6.13	1020
Alzheimer’s disease	M	65	6	C	08:50	6.88	1057
Alzheimer’s disease	M	65	5	C	05:50	6.36	1355
Alzheimer’s disease	M	65	5	C	07:20	6.47	1173
Alzheimer’s disease	M	87	5	C	06:10	6.14	1047
Alzheimer’s disease	M	67	5	C	04:10	6.40	1252
Alzheimer’s disease	M	70	6	C	04:50	6.95	1040
Alzheimer’s disease	M	82	5	C	05:15	6.34	1182
Nondemented control	M	73	0	O	24:45	?	1267
Nondemented control	M	71	1	O	07:40	6.20	1150
Nondemented control	M	87	1	A	10:20	6.32	1256
Nondemented control	M	80	0	O	07:15	5.80	1331
Nondemented control	M	84	1	A	05:35	6.98	1337
Nondemented control	M	82	1	O	05:10	6.75	1087
Nondemented control	M	78	1	O	< 17:40	6.52	1125

Medial temporal gyri from eight Alzheimer’s disease (AD) patients and seven age- and sex-matched controls were provided by the Netherlands Brain Bank. Braak stages based on neurofibrillary tangles were 5 or 6 in AD cases and 0 or 1 in controls (Braak & Braak, 1991). Braak stages based on amyloid plaques were C in AD cases and 0 or A in controls (Braak & Braak, 1991). Tissue preparation time from death is displayed as postmortem delay (PMD).

### Neuronal culture and transfection

Primary cultures of mouse cortical neurons were prepared from the brains of embryonic pups at day 16 as previously reported (Cho *et al*., 2014). In brief, the cerebral cortices were dissected from the embryonic brain and dissociated by trypsinization for 10 min at 37 °C. The resulting cell suspensions were resuspended in neurobasal medium supplemented with B27 (Gibco-BRL, Waltham, Massachusetts, USA) and plated onto poly-d-lysine-(Sigma, St. Louis, MO, USA) and laminin (Gibco-BRL)-coated plates or coverslips. Neurons were maintained at 37 °C in 5% CO_2_ for 12 days prior to chemical treatment. For transfection, primary cortical neuron cells were plated onto 24-well plates at a density of 4.8 × 10^6^ cells/plate and grown for 5 days before transfection. At DIV5, neuron cultures were transfected with DARPP-32-wild-type and DARPP-32-T153A mutant constructs using Lipofectamin 2000 (#11668-019; Invitrogen, Carlsbad, CA, USA) according to the manufacturer’s instructions. After 1.5 h, the transfected neuron culture medium was replaced with fresh conditioned medium containing antibiotics and the transfected neuron was treated with 1 μm oligomeric Aβ for 48 h.

### Drugs

Okadaic acid (10 nm; Boehringer Mannheim, Mannheim, Germany), calpain inhibitor-I (100 μm; Calbiochem), calpain inhibitor-III (100 μm; Calbiochem) and calpeptin (100 μm; Calbiochem) were added to neuronal cultures at the indicated final concentrations.

### Cell culture and Aβ treatments

SH-SY5Y, a human neuroblastoma cell line, was grown in Dulbecco’s modified Eagle’s medium (DMEM) supplemented with 10% foetal bovine serum (Hyclone, Logan, Utah, USA). Aβ oligomers were prepared as previously described (Song *et al*., 2014). Briefly, lyophilized Aβ peptides were dissolved in dimethyl sulfoxide, diluted in DMEM to a final concentration of 1 μm and incubated at 4 °C for 16 h. Prepared Aβ oligomers were added to SH-SY5Y cell lines for the indicated times.

### Site-directed mutagenesis

A threonine-to-alanine point mutation at residue 153 (T153A) of human DARPP-32 was introduced by Pfu Ultra HF (Agilent, Santa Clara, CA, USA) according to the manufacturer’s instructions and confirmed by sequencing. The sequences of the primers used were 5′-AGTCTGCTGG GCAAAAGGCA ACCTGTGGCC AGGGT-3′ (sense) and 5′-ACCCTGGCCA CAGGTTGCCT TTTGCCCAGC AGACT-3′ (antisense).

### Expression and purification of recombinant DARPP-32 proteins

cDNA encoding human DARPP-32 WT and the DARPP-32 T153A point mutant were cloned into the bacterial expression vector pGEX-4T-1 (GE Healthcare, Logan, Utah, USA) and transformed into *Escherichia coli* strain BL21(DE3) (Novagen, Darmstadt, Germany), respectively. For the expression of DARPP-32 WT and DARPP-32 T153A, transformed cells were grown in LB medium at 37 °C until an OD600 of 0.5 was reached. Protein expression was then induced by the addition of 0.5 mm isopropyl-β-d-thiogalactopyranoside (Sigma-Aldrich, St Louis, MO, USA) for 5 h at 28 °C. The recombinant proteins expressed were purified using GST•Bind Agarose Resin (Elpis Biotech) according to manufacturer’s instructions.

### Calpain cleavage assay

*In vitro* cleavage of recombinant DARPP-32 WT and T153A proteins by calpain was performed as previously described (Garg *et al*., 2011). Briefly, recombinant DARPP-32 WT or T153A (5 μg) was incubated with calpain-1 (Calbiochem) in reaction buffer (50 mm Tris–HCl, pH 7.5, 100 mm NaCl, 3 mm CaCl_2_, 2 mm DTT and 1 mm EDTA) for various times (1, 5, 10 and 30 min) with or without calpain inhibitors (100 μm zVAD and 100 μm calpeptin). After being incubated for the indicated times, the reaction mixture was mixed with an equal volume of 2 × SDS sample buffer and boiled for 10 min. Samples were subjected to SDS-PAGE followed by Coomassie staining or Western blotting with anti-DARPP-32 (Cell Signalling, Danvers, MA, USA) or anti-GST (Santa Cruz, Dallas, Texas, USA) antibodies.

### Western blotting

Cell or human brain tissue lysates were prepared with protein extraction solution (Pro-Prep; Intron, SungNam, Korea) in accordance with the manufacturer’s guidelines. Proteins were subjected to SDS-PAGE and subsequently transferred to PVDF membrane (Bio-Rad, Hercules, CA, USA) and blocked with 5% skim milk in TTBS buffer. Blots were incubated for 16 h at 4 °C with primary antibodies to DARPP-32 (1:1000; Cell Signaling, Danvers, MA, USA), phospho-DARPP-34 (1:1000; Cell Signaling), CREB (1:1000; Millipore, Darmstadt, Germany), phospho-CREB (1:1000; Millipore), c-*fos* (1:100; Santa Cruz), anti-HA (1:5000; Roche, Branchburg, NJ, USA), anti-spectrin (1:1000; Enzo Life Sciences, Farmingdale, New York, USA), anti-PP1 (1:200; Santa Cruz) and β-actin (1:1000; Sigma). The blots were washed in TTBS buffer, incubated with secondary antibodies for 1 h at 23 °C and visualized using enhanced chemiluminescence reagents (Thermo, Waltham, Massachusetts, USA).

### Quantitative analysis of neurite outgrowth

Primary neurons were transfected with DARPP-32 WT or T153A cDNA, which also independently express GFP. Low-resolution images (10 × magnification) of GFP-positive neurons (*n* = 100) were acquired from 20 to 65 different fields per sample. The neurite lengths and number of GFP-positive neurons in each image were measured using MetaMorph software (Universal Imaging Corporation, Marlow, Buckinghashire, UK).

### Quantitative real-time PCR

Human total RNA was purified from medial temporal gyri from eight AD patients and seven age- and sex-matched controls provided by the Netherlands Brain Bank (Table1) using a NucleoSpin RNA kit (Macherey-Nagel, Duren, Germany) according to the manufacturer’s protocol. Single-stranded cDNA was synthesized with SuperScript III Reverse Transcriptase (Invitrogen). Quantitative RT–PCR was performed using an iCycler (Bio-Rad). The primers used for RT–PCR were as follows: forward (binds to exon 1a, 5′-TTTTCATTTC TCACAAGGAC TGGGT-3′) and reverse (binds to exon 2, 5′-CTGGTGAGGA GTGCTCTGAG AGC-3′).

### Protein phosphatase 1 activity assay

SH-SY5Y cells expressing DARPP-32 WT or the T153A mutant were lysed with 1% Triton X-100 in PBS. Cell lysates were incubated with anti-PP1 antibody overnight at 4 °C and further incubated with protein G-sepharose (GE healthcare). Beads were washed three times with lysis buffer and incubated with 100 μm DiFMUP (fluorogenic PP1-specific substrate; Invitrogen) in reaction buffer (0.1 m sodium acetate, pH 5.0) for 30 min at RT. After incubation, supernatants were collected and fluorescence intensity was measured using a multiplate reader (Infinite M200PRO; TECAN, San Jose, CA, USA).

### Statistical analysis

Data are presented as means ± standard error of the mean (SEM) of at least three independent experiments and were analysed using Student’s *t*-test. *P* < 0.05 was considered statistically significant.
